# Critical Analysis of Pork QMRA Focusing on Slaughterhouses: Lessons from the Past and Future Trends

**DOI:** 10.3390/foods9111704

**Published:** 2020-11-20

**Authors:** Ammar Hdaifeh, Tahreem Khalid, Géraldine Boué, Enda Cummins, Sandrine Guillou, Michel Federighi, Vincent Tesson

**Affiliations:** 1 INRAE, Oniris, SECALIM, 44307 Nantes, France; ammar.hdaifeh@oniris-nantes.fr (A.H.); tahreem.khalid@oniris-nantes.fr (T.K.); geraldine.boue@oniris-nantes.fr (G.B.); sandrine.guillou@oniris-nantes.fr (S.G.); vincent.tesson@inrae.fr (V.T.); 2 Biosystems and Food Engineering, University College Dublin, Dublin 4 Belfield, Ireland; enda.cummins@ucd.ie

**Keywords:** food safety, QMRA, meat chain, meat processing, swine, pig

## Abstract

Foodborne microbial diseases have a significant impact on public health, leading to millions of human illnesses each year worldwide. Pork is one of the most consumed meat in Europe but may also be a major source of pathogens introduced all along the farm-to-fork chain. Several quantitative microbial risk assessment (QMRA) have been developed to assess human health risks associated with pork consumption and to evaluate the efficiency of different risk reduction strategies. The present critical analysis aims to review pork QMRA. An exhaustive search was conducted following the preferred reporting items for systematic reviews and meta-analyses (PRISMA) methodology. It resulted in identification of a collection of 2489 papers including 42 on QMRA, after screening. Among them, a total of 29 studies focused on *Salmonella* spp. with clear concern on impacts at the slaughterhouse, modeling the spreading of contaminations and growth at critical stages along with potential reductions. Along with strict compliance with good hygiene practices, several potential risk mitigation pathways were highlighted for each slaughterhouse step. The slaughterhouse has a key role to play to ensure food safety of pork-based products but consideration of the whole farm-to-fork chain is necessary to enable better control of bacteria. This review provides an analysis of pork meat QMRA, to facilitate their reuse, and identify gaps to guide future research activities.

## 1. Introduction

Foodborne microbial diseases have a major impact on human health worldwide as they result in a significant morbidity, mortality, and economic costs [1]. The World Health Organization (WHO) estimated, globally, 600 million (95% confidence interval (CI) 420–960 million) foodborne cases, and 420,000 (95% CI 310,000–60,000) deaths were transmitted by contaminated food in 2010, resulting in 33 million (95% CI 25–46 million) disability-adjusted life years (DALY) [2]. The severity of these diseases in humans varies from subclinical infection or mild symptoms to life-threatening conditions [2,3]. Amongst foods, meat and meat-based products are considered as one of the main vehicles of these pathogens. This food category accounted for almost 18% of total foodborne and waterborne outbreaks in Europe in 2018 [4].

According to Food and Agriculture Organization (FAO), pork is the most frequently consumed meat in the European Union (EU) in 2017 and it is the second-highest source of human meat borne disease in 2019 [4,5,6]. A report from European Food Safety Authority (EFSA) showed that pork meat and pork-based products are a source of foodborne illness in humans, particularly due to *Salmonella* spp., *Yersinia enterocolitica*, *Toxoplasma gondii,* and *Trichinella* spp. [7] and more recently, hepatitis E virus (HEV) [8]. EFSA also estimated in 2018 that salmonellosis was the second most reported zoonosis at the European level [4]. Moreover, pork meat is estimated to account for 20% of these foodborne salmonellosis cases [9].

The most frequent pathogen identified in pork meat is *Salmonella* spp., also considered as a major cause of foodborne illnesses [4,10,11]. Pork is one of the main reservoirs of *Salmonella* spp. that can be found in the digestive tract and the lymphatic tissue of the host and is a key vector of contamination as the live animals exhibit no symptoms of infection [7]. *S.* Typhimurium (classical and monophasic) as well as *S.* Derby were the most common serovar leading to human infections from pork meat in 2018 [4].

The pork meat chain consists of several steps from farm to human consumption. There are several possible routes of contamination including direct, inter-human, contact with infected pork, by contamination in the environment of the farm level, at slaughtering, during processing, retail, preparation and consumption. Thus, a risk-based food safety management approach was recommended in the last 20 years through international organizations like FAO, WHO and the Codex Alimentarius Commission (CAC), it consists of three components: risk assessment, risk management, and risk communication [12,13,14,15].

Microbial risk assessment (MRA) is defined as a scientifically based process consisting of four steps: hazard identification, hazard characterization, exposure assessment, and risk characterization. It is part of the risk analysis framework, as initially defined by FAO, WHO, and the CAC. According to the principles of risk assessment, as proposed by the CAC [12,13,14,15], a quantitative microbial risk assessment (QMRA) can be used to estimate the probability and severity of health risks following the ingestion of food-borne pathogens [16].

QMRA has proved, in previous decades, its usefulness to evaluate different control measures and guide risk managers to protect human public health [17,18]. A QMRA starts with the definition of a risk question formulated by risk managers and determines the QMRA’s objective by identifying the hazard, the product, the relevant steps (exposure scenario), and the targeted population forming the base for the construction of the QMRA model structure [16,19].

The objective of this work was to collect and analyze pork QMRA studies developed so far and to describe and identify critical steps along the pork production chain. A systematic approach was used to collect available pork meat QMRAs performed to date.

## 2. Materials and Methods

A literature search to collect and analyze risk assessment related to pork-based products has been done, using the PRISMA guidelines method [20,21] on Web of Science and Scopus databases.

The following search queries were used:

- Web of Science: TITLE: (pork* OR swine* OR porc* OR pig* OR cochon*) AND TITLE: (risk* OR risque* OR “risk assessment” OR aqr OR QMRA OR exposure OR “model$ing”);
- Scopus: TITLE (pork* OR swine* OR porc* OR pig* OR cochon*) AND TITLE (risk* OR risque* OR “risk assessment” OR aqr OR QMRA OR exposure OR modeling OR modelling).

The search was conducted in each database considering their respective starting year, 1956 for Web of Science and 1788 for Scopus to the date 11 Feb. 2020. A total of 4199 articles were collected, with the addition of 24 papers from the grey literature (mostly reports from European health agencies and WHO) and additional papers recommended by experts. Duplicates were removed and then articles were screened for titles, abstracts, and full-texts.

**Figure 1:**
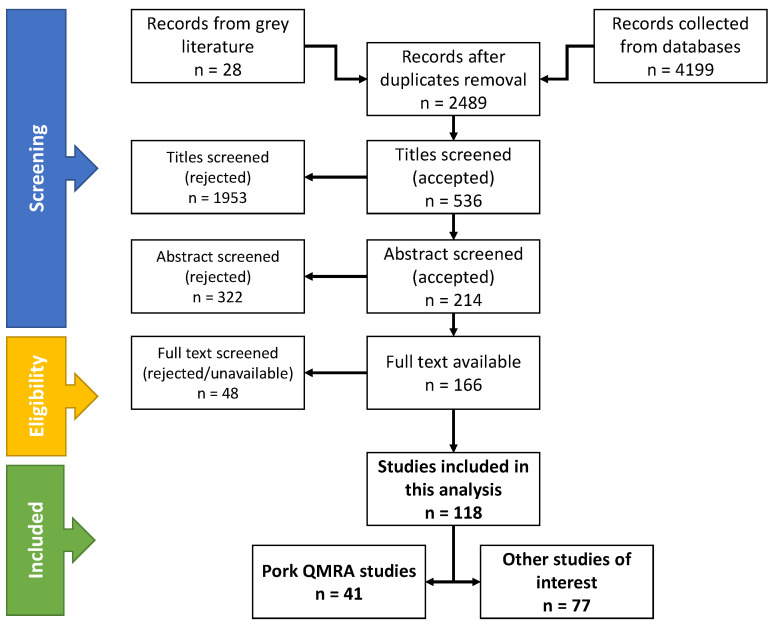
Flow chart of studies selected using the preferred reporting items for systematic reviews and meta-analyses (PRISMA) method. QMRA: quantitative microbial risk assessment.

During the selection process, only articles that met potential criteria were selected. A total of 118 articles were collected after screening through the preferred reporting items for systematic reviews and meta-analyses (PRISMA) method in which 42 studies specifically dealt with pork QMRA [22,23,24,25,26,27,28,29,30,31,32,33,34,35,36,37,38,39,40,41,42,43,44,45,46,47,48,49,50,51,52,53,54,55,56,57,58,59,60,61,62,63], and 77 additional studies focused on the following specific topics (Figure 1):

- Identification of risk factors [64,65,66,67,68,69,70,71,72,73,74,75,76,77,78,79,80,81,82,83,84,85,86,87,88,89,90,91];
- Evaluation of mitigation strategies along the meat chain [7,10,11,91,92,93,94,95,96,97,98,99,100,101,102,103,104];
- Predictive modeling with modelling of microbial growth, inactivation and survival [105,106,107,108,109,110,111,112];
- Estimation of microorganism prevalence at meat chain steps [82,89,113,114,115,116,117];
- Modeling of specific meat chain steps [118,119,120,121,122,123];
- Modeling of pathogen epidemiology and spreading during the meat chain [94,124,125];
- Expert analysis of sampling and mathematical methods [126,127,128];
- Qualitative or comparative risk assessment [129,130,131,132];
- Source attribution studies [133,134];
- Estimation of contamination levels in the slaughterhouse [135].

## 3. Results and Discussion

### 3.1. Analysis of Selected Studies

Studies covered four types of pork products: pork cuts, minced meat, fermented sausages, and carcasses (Table 1). Collected publications related mainly to European populations (*n* = 29) with a focus on Ireland (*n* = 6), Italy (*n* = 5), Denmark (*n* = 4), Belgium (*n* = 2), France (*n* = 1), Germany (*n* = 1), Poland (*n* = 1) and Spain (*n* = 1), while several studies were done at a European scale (*n* = 8). Besides that, some papers (*n* = 13) focused on other countries including Colombia, USA, Brazil, Thailand, Singapore, Argentina, Kenya, Australia and South Korea. Most of collected QMRA articles designed their study to cover the whole consumer population except for five that focused specifically on susceptible population to *Listeria monocytogenes* and *Toxoplasma gondii* contaminations [26,29,37,43,49].

The objective of these studies was to assess:

- Risks of illness associated with consumption of pork meat and preparations [22,23,24,26,27,28,29,30,32,33,34,35,36,37,38,39,40,42,43,44,45,46,47,48,49,50,51,52,53,54,55,56,57,58,59,61,62,63], sometimes based on review or meta-analysis [25,43];
- Exposure of consumers to pathogen due to the consumption of specific meat parts or the impact of a meat chain step [25,31,41,60];
- The effect of mitigation interventions or steps [23,26,33,34,36,37,41,45,46,47,50,51,56,57,58,59,61];
- Impact of data gaps or identification of risk factors [29,35,48,53,63].

#### 3.1.1. Pork Farm to Fork Chain

**Figure 2:**
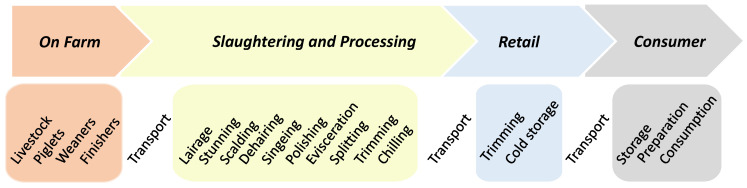
Farm-to-fork chain for pork meat as commonly considered by QMRA studies.

Pork undergoes a number of process and preparation operations along the meat chain in order to obtain the final product before it is consumed (Figure 2). This meat chain is constituted by four stages: on farm is where piglets are reared to obtain finisher pig; slaughterhouse corresponds to the place were pigs are processed to carcasses; retail assure carcass butchering and distribution to consumer while the final consumption part is were meat is cooked and consumed.

Almost all these operations can be considered as at risk for contamination of the product or the development of an existing contamination. Typically, three transportation steps occur, one for the animals from farm to slaughterhouse and the other transportation of product from slaughterhouse to retail and from retail to place of consumption.

#### 3.1.2. Pathogens Included in the Review of Pork QMRAs Studies

The QMRA studies dealt with three bacterial pathogens starting with *Salmonella* spp. [22,24,25,27,28,32,34,35,38,39,40,41,42,47,50,51,52,53,57,58,59,61,63,64], *Listeria monocytogenes* [37,49] and *Staphylococcus aureus* [30,48,62] including methicillin-resistant *Staphylococcus aureus* (MRSA). The remaining papers focused on three parasites (*Toxoplasma gondii* [26,29,43], *Trichinella spiralis* [36,54,55], *Taenia solium* [60]) and a virus (HEV [31]).

**Figure 3:**
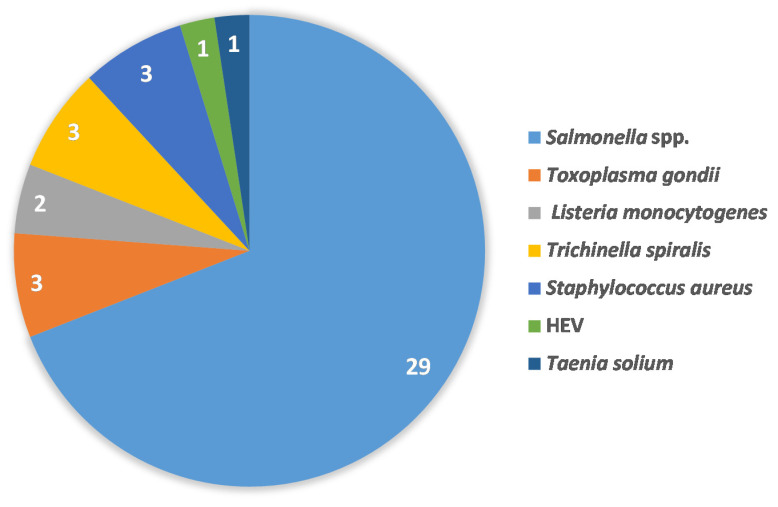
Pathogens included in the review. Numbers correspond to the count of the QMRA studies. HEV: hepatitis E virus.

According to collected QMRA papers, as shown in Figure 3, the main pathogen considered is *Salmonella* spp. compared to the other pathogens, supporting the significant importance of this pathogen.

Steps considered in QMRAs of *Salmonella* spp. were identified and listed in Table 2. A total of 14 publications covers the slaughterhouse steps during their risk assessment compared to the other parts of the meat chain.

Collected QMRA publications have shown that specific slaughterhouse interventions are more likely to produce larger reductions of human illness than interventions at the level of primary production [32,47,51,58]. For example, Swart et al. [58] showed that even a small number of infected pork at the farm-level could still lead to a high carcass prevalence at the end of the slaughter line due to the cross-contamination during the slaughter process and that post-evisceration intervention during processing appears the most effective means to mitigate human exposure.

In accordance with aforementioned analyses, *Salmonella* spp. contamination of pork carcasses during processing at slaughterhouse were investigated deeper. Each slaughterhouse step has been described with an emphasis on their respective impact on contamination prevalence and levels of the product (Table 3). 

### 3.2. QMRA and Slaughterhouse

#### 3.2.1. Transport and Lairage

When considered in QMRA, transportation and lairage step may cause an increase in the prevalence of infected animals [27,32,35,50,51,56,57,61,64]. For example, the transportation to lairage stage facilitates the mixing of animals and, hence, the cross-contamination of pigs from different regions, farms or pens [50]. It is a significant stage causing much stress for the animals, with noise, smells, and mixing with unfamiliar animals, changes in temperature, handling and environment [32,64]. During this stage, pigs can excrete *Salmonella* spp. in their feces, spread it in the environment and on their skin and cross contaminate other animals [27,32,35,50,51,56,57,61,64]. For example, Bollaerts et al. [27] found that after transport and lairage, 35.3% of the pigs are internally infected in the lymph nodes or/and the intestines and 20.6% of pigs are externally contaminated compared to 31.3% of seroprevalence of pigs at primary production. Pigs may experience the same stress factors during lairage as during transport due to the waiting period in the lairage for example. Delhalle et al. [32] showed that within 2–6 h of transport and lairage the number of animals that excrete *Salmonella* spp. can double. Lairage can act as a reservoir for pathogenic bacteria and there is evidence that longer holding times in the lairage can increase the risk of cross-contamination and rapid cross-contamination of pigs can occur [11].

#### 3.2.2. Slaughtering

As aforementioned, the slaughterhouse is one of the most documented parts of the meat chain with QMRAs [25,27,28,32,35,41,46,47,50,51,56,57,58,61,63,64]. Meat processing appears as a significant contributor to the final contamination of meat and meat products because it is a place that is favorable to the spread of *Salmonella* spp. through tools and equipment, and through inappropriate practices [11]. For example, the major sources of microbial contamination during processing are cross-contamination from the digestive tract of the animal during evisceration and the abattoir environment, i.e., equipment, machinery, and workers during carcass splitting and trimming [57,58]. Processing steps considered to cause contaminations during slaughtering are killing, dehairing, polishing, evisceration and splitting [25,27,32,58,63]. Moreover, around 20 to 25% of post-evisceration contaminated carcasses are estimated to having been contaminated through cross-contamination due to the slaughterhouse environment and staff practices [25].

##### Stunning, Killing, Bleeding

Upon entry to the slaughterhouse facility, the pigs are stunned using an electric shock [136] or a mixture of gas [58]. Stunning of animals is not considered as a step that will contribute to significant contamination to the animal [25]. Despite the lack of data concerning this step, a study showed that gas stunning (carbon dioxide) may lead to increased *Salmonella* spp. shedding through feces as a result of relaxed muscles, which is the opposite of the effects of the electrical stun [58]. Furthermore, pigs may have contact with each other and the slaughterhouse floor, potentially leading to cross-contamination [136].

##### Scalding and Dehairing

During the scalding step, carcasses are plunged into a hot water bath at 58–60 °C for a period of 5 to 6 min [50,58]. The main objective of this step is to loosen the carcass hairs in preparation for the following stage, which is dedicated to hair removal [58,136]. Dehairing takes place in a machine which consists of a rotating drum with internal extensions (e.g., metal brushes or flaps) [58,136]. Several studies showed an increase in prevalence and level of *Salmonella* spp. contamination at the dehairing step and a decrease of *Salmonella* spp. level at scalding [23,25,32,35,50,58]. The latter may be due to the heat stress induced to the bacteria, leading to the death of some with an observed decrease of skin prevalence from 31%, before bleeding, to 1% after scalding [98]. Additionally, the same study estimated the bacterial removal achieved by this operation to be about 3.5 log10 CFU/cm^2^. Nevertheless, Swart et al. [58] showed a potential risk of prevalence increase during scalding may occur due to *Salmonella* spp. organisms washed off the hide of contaminated carcasses while being able to survive long enough to subsequently contaminate the following carcasses to be scalded. Moreover, a study from Hill et al. [136] identified additional risks associated with bacteria enclosed in large fecal samples and baths at a temperature below 62 °C, respectively acting as a thermal protection and allowing for *Salmonella* spp. growth.

Concerning dehairing, two QMRAs [58,136] assumed that cross-contamination during dehairing will happen for both the carcass and dehairing machine as a consequence of the mechanical action of the machine, which is sufficiently strong to extrude some fecal material from the interior of the carcass. This may lead to an increase of both prevalence and levels of contamination [98].

##### Singeing

The singeing stage aims to burn any remaining hairs left after the dehairing phase [58,136]. To do so, generally, the pig carcass undergoes a heat treatment at high temperatures in a flaming oven, leading to the burning of hairs remaining after the dehairing step. Singeing is considered to be an effective inactivation step [11,27,32,58,64,136], able to lead to the complete removal of contamination from the carcass [94,98]. For example, Swart et al. [58] considered this step as the most effective stage for microbial inactivation with a time and temperature-dependent efficiency. A report from EFSA indicated that studies revealed a reduction of the total level of *Salmonella* spp. by approximately 1.5 to 3 log10 CFU on carcasses and a reduction of the prevalence on carcasses by 30 to 70% after the singeing step [11]. Efficiency of singeing may be reduced when the procedure is lightened, by either reducing its length or the temperature of flames, or when deep skin layers of the carcasses are highly contaminated [94]. A QMRA study estimated that the combination of all steps after killing of the pig up to singeing—included—resulted in a reduction of carcass prevalence from 49.7% to 2.3% [27]. 

##### Polishing, Washing

The polishing step is commonly done using a specific tunnel-shaped car-wash-like machine that will scrap the dried dirt and hairs remaining after singeing, more softly than for dehairing [136]. In some cases, this step can be manually done through classical washing of the carcass. Polishing is often associated with contamination of carcasses [25,27,32,58]. Similar to the dehairing phase, there is a risk of cross-contamination at this step and some fecal extrusion may occur [57,59]. Due to the configuration of the machines, these can spread bacteria onto the whole carcass and through the machine itself [32]. But according to Swart et al. [58] in this step, the amount of extruded fecal contamination is lower than in the dehairing phase. Moreover, after singeing, the level of contamination of the carcass is expected to be negligible [98].

However, if the singeing phase is not done correctly, additional contamination of the machine can occur from the carcass skin and this contamination can persist due to the difficulties to thoroughly clean the machine itself [94,98].

##### Evisceration

Evisceration is a delicate two-step stage, abdominal then thoracic cutting, which needs knowledge and experience from operators and it is one of the most critical processing steps, as often identified by collected papers [23,25,27,32,47,58]. It consists of the belly opening, the cutting of the breast bone, and the removal of the pluck (corresponding to the tongue, pharynx, tonsils, esophagus, trachea, heart, lungs, liver set).

Delhalle et al. [32] estimated the prevalence of *Salmonella* spp. for the carcass at evisceration to be 6.0% (SD ~ 1.3) while Swart et al. [58] estimate a prevalence between 10 and 40%. This prevalence can be increased during the operation through puncture of intestines or stomach during the belly opening causing potentially high contamination of the carcass. Contamination of the following carcasses is also possible as described by Bollaerts et al. [27]. According to a 2006 report from EFSA, it has been shown that a faulty evisceration step can be the cause for 55 to 90% of the number of carcasses contaminated with *Enterobacteriaceae* such as *Salmonella* spp. as well as of up to 90% of the contamination level of the carcass [11,94]. Moreover, a high number of *Salmonella* spp. positive samples were found in the evisceration environment of the slaughter line. However, the risk of a failure event at the evisceration stage is known and carefully considered so it remains infrequent (1–2%) [58]. In the end, evisceration is considered by several studies to increase the prevalence of contamination by between 0% to 7% [98].

##### Splitting, Trimming

The splitting step consists of halving the carcass, following the top-down axis, up to the neck using a mechanical saw. The carcass is then dressed to remove kidneys along with some fat and then it is trimmed to remove any remaining visible contamination. 

Contamination during splitting and trimming is linked with cross-contamination from the splitting machine to the carcass [25,57,58] with a probability of cross contamination up to 50% [25]. Barrón et al. [25] estimated, through a meta-analysis-guided QMRA, the mean prevalence of carcasses contaminated by *Salmonella* spp. after splitting and trimming to be 14.2% compared to 11.9% after evisceration. Compared to splitting, Swart et al. [58] reported that trimming has no significant effect since it consists of removal of detected fecal contamination (from polishing or belly opening). This considers the fact that actions are done using a sterilized knife between cuts and that cuts are done to remove a large portion of meat around the contamination area. Concerning carcass trimming especially, note that operators following good hygiene practices should not use the same knife for different carcasses without decontamination of the knife. Further, even with this intervention, contamination can still remain on the knife. In the EU for example, this decontamination consists in a bath in water at a temperature ≥82 °C. This helps to achieve a sufficient bacterial removal but only if the knife is cleaned of any remaining fat or protein as these organic matters may protect the bacteria from heat [104].

##### Chilling

At the end of the processing chain, the chilling step is considered as a crucial step to minimize any potential increase in the prevalence of contamination on the carcass/product before leaving the slaughterhouse [25,27,32,35,41,51,58,63,64]. During this step, the carcass temperature should decrease from 39–42 °C to 7 °C within 24 h [11].

It is also considered as an inactivation step due to the difficult recovery of freeze- or chill-injured cells [27,35]. Gonzales-Barrón et al. [41] suggested, based on a meta-analysis, that chilling almost halves the occurrence of *Salmonella* spp. in pork carcasses. Nevertheless, the majority of authors consider that chilling of carcasses has no significant effect on their bacterial growth [27,32,35,41,58]. For example, Swart et al. [58] showed that *Salmonella* spp. does not grow, nor decrease when keeping the half-carcasses cooled at 4 °C for an extended period, until the carcasses are transported to the cutting plant. On the other side, Fajardo et al. [35] showed an increase in the prevalence of *Salmonella* spp. during chilling in-plant, characterized by lack of HACCP (hazard analysis critical control point) plans implementation in the slaughterhouse.

However, it is considered that the effect of the chilling step on contamination levels is still the subject of debate, with issues concerning the impact of a plethora of factors such as total sample size or size of swabbed area [10,11,41] along with the effect of the carcass characteristics itself (weight and fat cover) or the chilling procedure [98]. This leads to vast variability of results and, in some circumstances, may even result in an overestimate of risks due to conservative assessment of the stressed state of bacterial cells [10]. Moreover, it is increasingly considered that surviving bacteria can begin growth again during cold storage, especially following slow chilling methods [11,98].

#### 3.2.3. Overall Impact of Slaughterhouse Operations

Operations at the slaughterhouse are of major concern as it includes the most critical contamination steps, e.g., evisceration or splitting [10,11,23,34]. By lowering, as much as possible, the contamination level and prevalence, it ensures negligible bacterial growth in the latter stages of the chain at both retail and home storage and also following cooking. 

Nevertheless, implementation and monitoring of good operational and hygiene practices during each step is essential, but may not always be sufficient to achieve proper meat safety objectives [10,11,23].

### 3.3. Mitigation Measures

#### 3.3.1. Lairage Length

When pigs are unloaded from the transport trucks, animals are not immediately stunned/killed. According to the speed of the processing line, animals may spend several hours in boxes previously occupied by another animal, which were potentially contaminated. During this waiting time, the animal can be stressed and shed higher levels of *Salmonella* spp. than usual through its feces. Bollaerts et al. [27] developed a probabilistic risk assessment model for farm-to-fork pork (METZOON) and modelled the effects of 14 intervention scenarios to reduce human salmonellosis cases. Reductions of *Salmonella* spp. prevalence, both internal and external, at the lairage were estimated to be between 10 to 75%. Tested scenarios involving a reduction of infected animals were categorized as ‘less effective’ while scenarios involving a reduction of externally contaminated animals were considered as ‘not effective’, relative to other scenarios considered, e.g., reduction during polishing, evisceration or chilling. On the other side, Delhalle et al. [32] tested alternative scenarios corresponding to potential mitigation strategies revealing the most efficient intervention action at lairage for reducing human salmonellosis risk as well as evisceration and chilling steps. Reduction of *Salmonella* spp. prevalence in pigs entering lairage by 25%, 50% and 75% was modelled, yielding a predicted relative reduction in human salmonellosis cases ranging from 24.7%, 33.8 and 84.2%, respectively.

#### 3.3.2. Slaughterhouse

To mitigate the risks described above, risk managers and authorities have worked on the impact of implementing measures along the meat chain. Measures can be implemented throughout the entire chain, but mitigations at critical vulnerable steps may be the most effective, including for example lairage, evisceration and chilling steps. Some studies concluded that decreasing on-farm *Salmonella* spp. prevalence has a much smaller effect than a similar reduction in prevalence during slaughter and processing along with a unfavorable benefit/cost ratio and almost no long-term persistence [23,34,47,51,91]. Other measures that are considered as essential are frequent cleaning of the processing line or separation of the line into a “slaughter area” and a “carcass area” with the belly opening step acting as a boundary [34]. Besides these measures, more specific ones can be implemented (Table 4). Of course, combinations of mitigation measures applied at each level of the meat chain remain the most efficient ones [23].

#### 3.3.3. Logistic Slaughter 

A risk mitigation intervention can be applied on the entire processing chain rather than a specific step. Logistic slaughter is an option for reducing the pathogen load on the carcasses of slaughtered pigs [10,11,23,45]. Logistic slaughtering is done by the separate slaughtering of *Salmonella*-negative, or low prevalence, herds before positive herds or high-risk herds (high shedding) throughout the day [10,23,45]. This way, the cross contamination that could happen during the random processing of batches should be theoretically reduced to its minimum.

To be as efficient as possible, logistic slaughter needs the implementation of a separation of the different herds during animal transport, unloading and lairage to reduce the risks of stress-induced excretions. This way, risks of subsequent cross-contamination, and thus the contamination of carcasses during the processing chain, are mitigated. To a certain extent, pushing forward the segregation up to the slaughtering and processing chains according to the herd status for *Salmonella* spp. may be the best way to implement logistic slaughtering [11].

Logistic slaughtering has limitations regarding its general implementation. Firstly, it is really efficient only if a range of factors are overseen: continuous and close monitoring of the *Salmonella* spp. status of herds during pre-slaughtering steps, rigorous and frequent application of hygiene measures during post-farm steps, i.e., trucks, unloading docks, lairage pens [23,45]. In addition, Hill et al. estimated, using a farm-to-fork risk assessment, a negligible impact of logistic slaughtering intervention on the prevalence of contaminated carcasses and subsequent salmonellosis incidence [45]. In the same vein a report from FAO-WHO considered, after systematic review, that this intervention could not be recommended considering the lack of consistent evidence [10]. Nevertheless, the report insists on the fact that logistic slaughtering would reduce *Salmonella* spp. levels during post-farm steps and that, in all occasions, pigs from high-prevalence herds should be handled with caution.

#### 3.3.4. Spray Scalding

Spray or steam scalding is an alternative to the classical tank scalding method to avoid the contamination issues associated with temperature and time abuses during scalding of carcasses [34,68]. To do so the carcasses are cleaned by applying a uniform powerful steam spray in order to clean and loose the pig’s hairs without having to reuse the now contaminated water for the following carcass to be processed.

Very few data on the impact of this method are available as this technology is not widely deployed. However, an analysis of the risk factors for *Salmonella* spp. for 10 Belgian pork slaughterhouse through the use of a regression models revealed that the use of steam scalding was negatively associated with a risk of salmonellosis for consumers (*p* = 0.033) [68].

#### 3.3.5. Evisceration-Specific Procedures and Post-Evisceration Inspection

Concerning the impact of evisceration on carcass contamination, failures during operation are not a common event but have to be considered. As such, simple procedures can be easily implemented for reasonable efficiency. In order to avoid spilling of the gastrointestinal contents from inside the carcass, one procedure commonly implemented is the sealing of the rectum within a plastic bag, leading to the collection of all dirt spilled during this operation [94]. In addition, an EFSA report identified another solution by the slowdown of this specific step, through the use of parallel branches, in order to reduce the risk of mishandling [11]. 

A study conducted by Snary et al. [57] showed the importance of a post-evisceration control step to take highly contaminated carcasses off the line. This way, subsequent carcasses have reduced risk of cross-contamination and the level of *Salmonella* spp. on the carcass at the end of processing will be reduced.

#### 3.3.6. Double Singeing

In some processing lines a second singeing can be done after the polishing step [11,23,32,34,68]. In this case, the flames temperature of the first singeing is slightly reduced to avoid meat alteration due to the second flaming. A report from French Food Safety Agency (Afssa, former name of Anses) showed the efficiency of double singeing on the contamination levels on the surface of carcasses, with a 0.5 decimal reduction [64]. To achieve this level of reduction, the flame should be able to reach all parts of the surface of the carcass [11,34].

#### 3.3.7. Hot Water and Organic Acid Washes

Final washing of the carcass, between its splitting and chilling, is considered one of the best ways, after chilling, to reduce the level of contamination of the carcass before its processing [25,136]. Indeed Barron et al. [25] reinforced, through regression sensitivity analysis, the importance of final rinsing (r = −0.382) and chilling (r = −0.221) as stages that contribute to a reduction of the prevalence of *Salmonella* spp. on the final product. Concerning the impact on contamination level, a report from WHO indicated that hot water rinsing can achieve a reduction of 2 log10 CFU/cm^2^ [10], while an Anses report estimates a reduction between 2.5–3.7 log10 CFU [23]. In contrast, a report from EFSA showed that final washing using cold water increases the bacterial level by between 3.6 to 3.8 log10 CFU/cm^2^ [11]. The WHO report also advises to attain a temperature of at least 70 °C at the carcass surface through the hot water rinsing to achieve maximum reduction.

Organic acid washing, using either lactic, acetic, citric or fumaric acids, is known to reduce the *Salmonella* spp. prevalence from 7% to 2% [10]. Compared to cold water rinsing, the use of organic acids results in a wide range of *Salmonella* spp. level reductions. Therefore, it is considered that this method can achieve realistic reductions between 0.5 to 1.0 log10 CFU.cm^−2^ [10]. At the carcass scale, the total reduction achieved is considered between 2–3 log10 CFU for the vegetative forms of the pathogens, including *Salmonella* spp. [11]. To be able to achieve the maximum microbial level reduction, washing should be done for the entire carcass, uniformly, using a suited combination of concentration, time, contact duration and temperature. Thus, concentrations should be considered as both plant- and compound-specific while considering the consecutive steps, if followed by rinsing, the contact time of acids may be considered [10]. In addition, it appears that the solution temperature also plays a significant role, ensuring safety without alteration of the meat [34].

Drawbacks identified concerning enhanced rinsing methods include the potential positive selection of resistant strains when shifting from laboratory-scale experimentations to commercial scale applications due to the initial contamination level on the carcass, difficulties to achieve uniform solution spreading over the entirety of every processed carcasses and the limited reductions achieved [11,101,102,103]. Rajkovitc et al. described for example how acid-tolerant *L. monocytogenes* strains might be selected through several lactic acid rinsing cycles [102]. Other concerns include the activation of virulence factors for stressed cells and facilitated growth due to the destruction of the meat background microflora [11]. Moreover, if done incorrectly, acid washing can induce an increase of the prevalence as identified by Fajardo-Guerrero [35]. In addition, the use of chemicals for meat sanitation is prohibited for meat to be sold in European countries [11,23]. 

## 4. Conclusions

Foodborne diseases originating from meat consumption represent a significant proportion of all foodborne diseases reported in the world, with a large number of them caused by pork meat. The pathogens involved in these food-borne diseases contaminate meat all along the meat chain, passing through the different stages of the chain. To identify the knowledge gaps concerning factors influencing contamination events and changes, this study aimed to synthetize available data through a systematic review of published QMRAs done on pork meat contamination.

Through the analysis of this collection of scientific papers it emerged that *Salmonella* spp. was the main pathogen assessed by authors and that the meat chain stage that was the most monitored was the slaughterhouse. These studies emphasized the fact that, during the slaughterhouse stage, there is a set of steps that may be considered as the main control points to prevent contamination, either in terms of increasing prevalence or pathogen concentrations. The principal steps are dehairing, polishing and evisceration [25,27,32,58,63]; with the last considered to be the main source of contamination of carcasses before chilling [11,94]. On the other side, several studies agree that the slaughterhouse stage is where interventions are the most efficient to reduce the risk of illness for consumers. The main inactivation steps identified were scalding, singeing, washing and chilling; with singeing being considered as the only step which can totally remove contamination from a carcass [94,98]. However, gathered data highlighted the importance of good process and hygiene practices. Thus, poor machine cleaning procedures or procedure alterations may lead to the dispersion of contamination within the machines and throughout the processing line or an opportunity of growth for stress-damaged cells [11,25,27,57,58,94,98,101,102,103,136]. 

Thus, in addition to these practices, several points of concern were put forward as to be addressed in the nearest future. Procedures and equipment improvements were highlighted as risk mitigation measures. Better consideration of the infection status and welfare of pigs concerning their transport and slaughtering, improvement of decontamination steps was also underlined through this analysis. Despite all these enhancements, some uncertainties lie within procedures with unexpected flaws or a lack of data waiting to be addressed. For example, the possibility of contamination of the lungs of the pig, here estimated for *P. multocida*, with contaminated scalding water, may be hazardous at the pluck-removal in the evisceration stage and lead to carcass contamination [34]. Another example could be cross-contamination when using gas stunning of batches of pigs, which is not usually modelled [58,136]. Moreover, a point usually underestimated is that *Salmonella* spp. contamination rates and the possibility of cross-contamination are known to be slaughterhouse-specific and depends on adherence to procedures, cleaning practices, farm-specific prevalence of supplied herds and plant size [92]. This leads to the necessity to adapt every mitigation intervention to the specific constraints of each slaughterhouse environment. On a more global scale, further consideration for QMRAs covering the entire farm-to-fork chain may enable better prioritization of actions that can be applied along the entire chain and provide a more holistic view of different mitigation possibilities [34,92]. Enhanced management of finishing pigs with careful monitoring of *Salmonella* spp. status [11,34,94] along with feed and water acidification to reduce contamination levels in feces [10,11,34] are examples of what can be done at the farm level. Concerning the consumer stages, irradiation of minced meat is considered by FAO/WHO [10] but cannot be applied for meat sold in Europe. With respect to the cold chain, HACCP implementation and improved consumer education are also publicized [10,11]. 

In conclusion, where food safety management was previously based on constant monitoring of the production and post-crisis reaction, the shift to a risk-based management food system has helped to identify the procedural flaws were risk may be amplified. Thereafter, QMRA has helped to identify and prioritize interventions considering the entire farm to fork chain. Nevertheless, even if a microbial hazard is difficult to remove, efforts should be made throughout the whole production chain, with a higher priority for interventions at the slaughterhouse as combination of mitigation intervention applied here appear as the most efficient ones for reducing the illness risk for consumer [23,34,47,51,91].

This study reviewed existing scientific and grey literature regarding quantitative risk assessment of pork products. The PRISMA methodology was applied to identify relevant scientific studies and a critical analysis of available studies was conducted. This study provides insights into the important steps in the farm-to-fork pork production chain and presents a unique critical evaluation of each stage with regards to potential contamination events. This study highlights available evidence on mitigation strategies for the management and control of pathogens in the pork chain and hence facilitates better food safety management for the benefit of all stakeholders. 

## Figures and Tables

**Table 1:** Summary of pork quantitative microbial risk assessment (QMRA) models collected.

Pathogen	Product	Country, Year	Population	Objective	References
*Salmonella* spp. *(n = 29)*	Pork products	France, 2018	All	Risk assessment; intervention ranking	[23]
		U.S.A, 2007	All	Risk assessment; intervention ranking	[50]
		E.U., 2010	All	Risk assessment; intervention ranking	[34]
		E.U., 2016	All	Risk assessment, analysis of risk mitigation measures	[45,46,56,57,58,59,61]
		Italy, 2004	All	Risk assessment for consumption of Italian regional meat products	[38]
	Sausages	Denmark, 2002	All	Risk assessment to design monitoring and management interventions according meat contamination.	[22]
		Brazil, 2011	All	Risk assessment after barbecue cooking	[52]
		Ireland, 2010–2012–2015	All	Chilling-focused risk assessment	[24,39,40,42]
	Pork preparation	Thailand, 2004	All	Risk assessment; correlation study between factors affecting exposure to *Salmonella* spp. and illness risk	[53]
	Minced Pork	Belgium, 2009	All	Risk assessment for household consumption	[27]
				Risk assessment for food safety improvement	[32]
	Pork meat	Colombia, 2020	All	Comparative risk assessment to evaluate the impact of HACCP implementation in two slaughterhouses.	[35]
	Burgers	Australia, 2018	All	Commissioned risk assessment for risk management consulting	[44]
	Ground pork	USA, 2019	All	Risk assessment, identification of critical control points	[63]
	Carcasses	Ireland, 2009–2013	All	Meta-analysis supported exposure assessment carcasses	[25]
				Assessment of the impact of chilling on *Salmonella* spp. occurrence in carcasses	[41]
		Denmark, 2017–2008–2016	All	Hygiene-performance based (*E. coli* levels) risk assessment	[28]
				Assessment of actual herd classification schemes, procedures comparison	[47]
				Assessment of interventions oncarcasses	[33]
		USA, 2005	All	Benefit/cost process and interventions assessment	[51]
*Staphylococcus aureus* *(n = 3)*	Pork meat	U.S.A, 2014	All	Risk assessment, identification of data gaps and risk factors	[48]
		Korea, 2009	All	Pathways to humans for MRSA ^1^	[62]
	Carcasses	Germany, 2014	All	Risk assessment for MRSA	[30]
*Listeria monocytogenes* *(n = 2)*	Ready-to-eat	Italy, 2010	Susceptible	Risk assessment in support of risk management decisions	[49]
		Spain, 2010	Susceptible	Risk assessment, intervention assessment	[37]
*Trichinella spiralis* *(n = 3)*	Carcasses	Poland, 2018	All	Risk assessment considering housing condition and meat inspection	[36]
	Pork meat	Singapore, 2009	All	Risk assessment for imported chilled meat	[54]
	Sausages	Argentina, 2016	All	Risk assessment	[55]
*Toxoplasma gondii* *(n = 3)*	Pork meat	Italy, 2018	Sensitive population	Risk assessment of fresh or frozen pork or cattle meat	[26]
			All; pregnant women	Risk assessment for Italian population, sensitivity analysis	[29]
	Fresh pork	U.S.A, 2017	All; pregnant women	Review-based risk assessment	[43]
*Taenia solium* *(n = 1)*	Pork meat	Kenya, 2017	All	Exposure assessment for western Kenyan population	[60]
HEV ^2^ *(n = 1)*	Organs	Italy, 2018	All	Exposure assessment for organs of food safety interest, impact of data gaps	[31]

^1^: MRSA: methicillin-resistant staphylococcus aureus; ^2^: HEV: hepatitis E virus.

**Table 2:** Meat chain steps considered in collected pork QMRAs for *Salmonella* spp.

Reference	Farm Level	Slaughterhouse	Retail	Consumption
Anses, 2018 [64]	✔	✔		✔
McNamara et al., 2007 [50]	✔	✔	✔	✔
EFSA, 2010 [34]	✔	✔	✔	✔
Snary & Swart, Vigre and Simons & Hill et al., 2016 [46,56,57,58,59,61]	✔	✔	✔	✔
Giovanini et al., 2004 [38]			✔	✔
Alban et al., 2002 [22]			✔	✔
Mürmann et al., 2011 [52]			✔	✔
Gonzales-Barron and Butler, 2015; Gonzales-Barron et al., 2010, 2012 [24,39,40,42]			✔	✔
Osiriphun et al., 2004 [53]				✔
Bollaerts et al., 2009 [27]	✔	✔	✔	✔
Delhalle et al., 2009 [32]	✔	✔	✔	✔
Fajardo-Guerrero et al., 2020 [35]	✔	✔		
Zhang et al., 2019 [63]	✔	✔	✔	✔
Gonzales-Barron et al., 2009 [25]		✔		
Gonzales-Barron et al., 2013 [41]		✔		
Bollerslev et al., 2017 [28]		✔		
Hurd et al.,2008 [47]		✔		
Duarte et al., 2016 [33]		✔		
Miller et al., 2005 [51]	✔	✔		
**Total**	**9**	**14**	**10**	**12**

**Table 3:** Slaughterhouse steps considered as impacting level or prevalence of contamination in collected pork QMRAs for *Salmonella* spp. **−**: steps considered as decreasing concentration or prevalence; **+**: steps considered as increasing concentration or prevalence; **+/−**: steps considered as both increasing and decreasing concentration or prevalence.

References	Lairage	Stunning	Scalding	Dehairing	Singeing	Polishing	Evisceration	Splitting	Trimming	Chilling
Anses 2018 [64]	**+**				**−**		**+**			**−**
McNamara et al., 2007 [50]	**+**		**−**							
EFSA 2010 [34]	**+**		**−**	**+**	**−**	**+**	**+**	**+**	**−**	**−**
Snary, Swart, Vigre, Simons, Hill et al. 2016 [46,56,57,58,61]	**+**		**+**	**+**			**+**			**−**
Bollaerts et al., 2009 [27]	**+**	**+**			**−**	**+**	**+**			**−**
Delhalle et al., 2009 [32]	**+**		**−**	**+**	**−**	**+**	**+**			**−**
Fajardo-Guerrero et al., 2020 [35] ^a^	**+**		**−**				**−**			**−**
Zhang et al., 2019 [63]									**+**	
Barron et al., 2009 [25]		**+**		**+**	**+**	**+**	**+**	**+**	**+**	**−**
Gonzales-Barron et al., 2013 [41]										**−**
Bollerslev et al., 2017 [28] ^a^										
Hurd et al., 2008 [47]							**+**			
Duarte et al., 2016 [33] ^a^										
Swart et al., 2016 [58]			**+/−**	**+**	**−**	**+**	**+**	**+**	**−**	**−**
Miller et al., 2005 [51]	**+**	**−**	**−**	**−**	**−**	**−**	**−**	**−**	**−**	**−**

^a^: Slaughterhouse was only considered as source for input data for the model (prevalence and/or level of contamination at plant exit).

**Table 4:** Summary of risk mitigation measures identified for slaughterhouse implementation through pork QMRAs.

Step	Risk Factors	Risk Mitigation Measures	References
Transport, lairage	Soiled environment	- Short transport/lairage time - Separation of pig lots - Separation of trucks delivering - Regular cleaning and bacteriological inspection of truck/lairage pens	[11,32,50,51,57,58,64]
Scalding, dehairing	Water temperature Duration Unclean water	- Water temperature of 61–62 °C/8 min or 70 °C/2–3 min - Spray/steam scalding	[10,25,34,50,58]
Singeing	Temperature Length	- Temperature of 800–1000 °C - Double singeing	[11,23,32,34,58]
Polishing, washing	Cross-contamination (machines)	- Equipment cleaning	[11,25,32]
Evisceration	Gut rupture Cross-contamination (handler, knives)	- Colon and rectum pre-sealing - Pre-evisceration hot water washing	[11,25,27,32,47,58,64]
Splitting, trimming	Cross-contamination (machine)	- Regular cleaning (machine)	[25,58]
Chilling	Temperature Duration	- Extended chilling time at 4 °C - Bring internal temperature to 20–25 °C within 2–3 h post-slaughter	[11,58]
Logistic slaughter	Cross contamination (processing line)	- Harvest *Salmonella*-negative animals first - Trucks/docks/lairage pens disinfection - Dedicated processing lines	[10,11,64,136]
Washes containing organic acids	Level of contamination	- Concentration - Contact time - Temperature	[10]
